# Atypical bilateral ventilation/perfusion mismatches in an asymptomatic patient suffering from metastatic thyroid cancer

**DOI:** 10.1186/s41824-021-00120-3

**Published:** 2021-12-20

**Authors:** David Kersting, Christoph Rischpler, Till Plönes, Clemens Aigner, Lale Umutlu, Ken Herrmann, Hubertus Hautzel

**Affiliations:** 1 Department of Nuclear Medicine, University Hospital Essen, West German Cancer Center (WTZ), University of Duisburg-Essen, Hufelandstrasse 55, 45147 Essen, Germany; 2 Department of Thoracic Surgery and Thoracic Endoscopy, University Medicine Essen - Ruhrlandklinik, West German Cancer Center (WTZ), University of Duisburg-Essen, Essen, Germany; 3 Department of Diagnostic and Interventional Radiology and Neuroradiology, University Hospital Essen, West German Cancer Center (WTZ), University of Duisburg-Essen, Essen, Germany; 4 German Cancer Consortium (DKTK), Partner Site University Hospital Essen, Essen, Germany

**Keywords:** Perfusion scintigraphy, MAA SPECT, Pulmonary embolism, Thyroid cancer

## Abstract

**Background:**

Pulmonary embolism is indicated by ventilation/perfusion (V/P) mismatches in ventilation/perfusion scintigraphy. However, other pathologies may also evoke segmental or lobar mismatches. Thus, diagnosis can be difficult in asymptomatic patients with equivocal clinical presentation.

**Case presentation:**

We present a case of multiple bilateral pulmonary ventilation/perfusion mismatches in a poorly differentiated thyroid cancer patient. Exact diagnosis was difficult, as the patient was asymptomatic and pulmonary embolism is commonly unilateral in tumour patients and not typical for thyroid cancer. External pulmonary artery compression by aortic aneurysm, multiple metastases or additional bronchopulmonary malignancies were considered as differential diagnosis. After unilateral pulmonary and hilar metastasectomy, perfusion normalised on the operated side. Pulmonary perfusion defects due to pulmonary artery compression by hilar metastases were finally diagnosed. Pulmonary embolism was deemed unlikely due to the left-sided post-operative normalisation, persistence of right-sided V/P mismatches, and the lack of clinical symptoms.

**Conclusion:**

Pulmonary artery compression may mimic pulmonary artery embolism in lung perfusion scintigraphy and should be considered in bronchopulmonary tumour patients with hilar metastases and unilateral ventilation/perfusion mismatches affecting a complete lobe or even lung. Following the presented case, also bilateral segmental and subsegmental mismatches in patients with hilar metastases from non-bronchopulmonary cancer entities should be carefully evaluated.

## Background

Segmental or subsegmental ventilation/perfusion (V/P) mismatches in ventilation/perfusion scintigraphy indicate pulmonary embolism (PE) with high sensitivity and specificity (Bajc et al. 2019). However, other pathologies such as vasculitis or congenital vascular abnormalities may also evoke segmental or lobar mismatches (Bajc et al. 2019). Thus, diagnosis can be difficult in cases with equivocal clinical presentation.

PE due to venous thromboembolism (VTE), mostly diagnosed in routine staging CT, is a frequent and challenging complication in cancer patients (Donadini et al. 2014). However, in thyroid cancer patients only little data are available on VTE, which may be unlikely in this patient cohort (Ordookhani et al. 2018). Differential diagnoses are perfusion defects due to external compression by aortic aneurysm (Makis and Derbekyan 2012), metastases, or bronchopulmonary tumours (Cei et al. 2004). Additionally, vasculitis and congenital pulmonary vascular malformations (Bajc et al. 2019), pulmonary arterial hypertension (Chan et al. 2018), and extensive emphysematic bullae (Reinartz et al. 2004) can be considered. Recently, a unilateral perfusion defect caused by pulmonary vein stenosis after pulmonary vein isolation has been reported (Kind et al. 2021).

## Case presentation

**Fig. 1:**
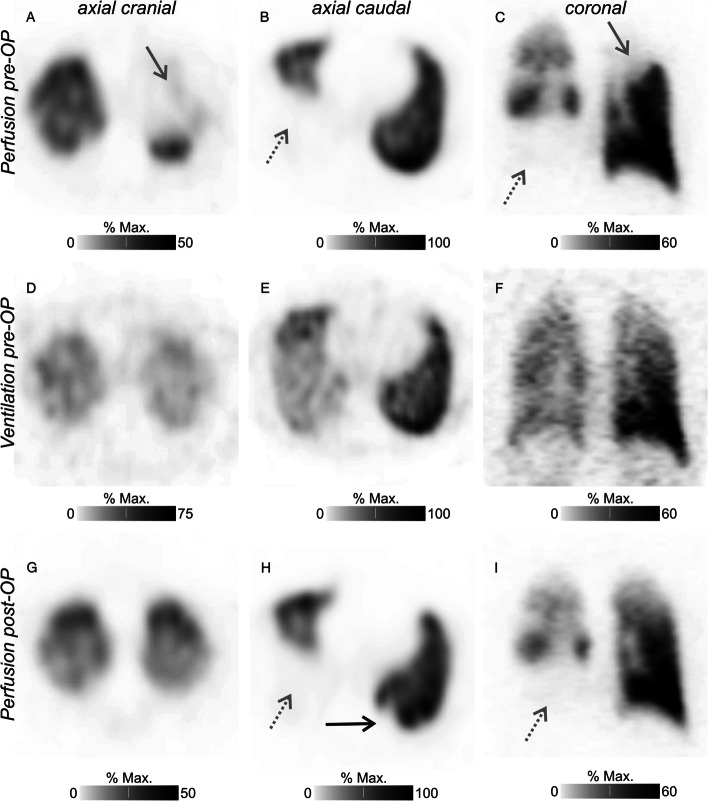
Preoperative perfusion (**A**–**C**), corresponding preoperative ventilation (**D**–**F**) and post-operative follow-up (**G**-**I**) perfusion SPECT images. Perfusion defects are indicated by solid (left superior lobe segments S1/2L and S3L) and dashed grey arrows (middle lobe and right inferior lobe). Left-sided pulmonary perfusion was normalised after surgery; a subsegmental probably post-surgical defect is indicated by a black arrow (left inferior lobe segment S10L)

A 38-year-old man was referred to our clinic 6 months after initial diagnosis of poorly differentiated thyroid carcinoma (TNM: pT3a pN1b pM1). Prior to resection of radioiodine-negative pulmonary metastases, perfusion scintigraphy was requested for preoperative risk evaluation, as sequential bilateral metastasectomy was planned. In addition, the patient showed a reduced lung reserve in pulmonary function testing (maximum vital capacity 3.9 L, 80% of the nominal value; forced vital capacity 3.5 L, 69% of the nominal value; forced expiratory volume in 1 s 2.9 L, 71% of the nominal value). Thoracic SPECT imaging after application of ^99m^Tc-macro-aggregated albumin (MAA) demonstrated multiple bilateral pulmonary (multi-)segmental (right lower lobe, middle lobe, left upper lobe) and subsegmental (e.g., right upper lobe) perfusion defects (Fig. 1A–C). Subsequently performed ventilation SPECT using ^99m^Tc-Technegas did not show any impairment (Fig. 1D–F). The V/P mismatches were highly suspicious of multifocal PE. However, the patient did not report symptoms of PE. Both blood panel and spirometry did not reveal any pathological findings; oxygen saturation was 98.5%. The clinical pretest probability for PE was low with a Wells score of 1 and a revised Geneva score of 2. In combination with the clinical findings, PE was deemed unlikely; therefore, no anticoagulation treatment was initiated. The planned resection was extended to left pulmonary and left hilar metastasectomy: 8 pulmonary wedges containing metastases (S3, S4, upper lobe/lingula, S10, lower lobe/diaphragm surface) and an aortopulmonary window lymph node metastasis were resected. Another hilar metastasis was not removed because of its close proximity to the upper lobe artery. As subsequent right-sided metastasectomy was considered, a follow-up perfusion SPECT was performed 2 weeks after left-sided metastasectomy. Left-sided pulmonary perfusion was normalised apart from one newly occurring subsegmental defect in left inferior lobe segment S10 (Fig. 1H) interpreted as post-surgical (compare Fig. 1B and H), while perfusion defects on the right side that had not underwent surgery remained unaltered (Fig. 1G–I). Within 1 week after the second perfusion SPECT, 4 pulmonary wedges containing metastases (upper lobe, lower lobe fissure, S6, S10) were resected on the right side. Mediastinal metastases were not resected since they were in close proximity to central vessels and bronchi.

**Fig. 2:**
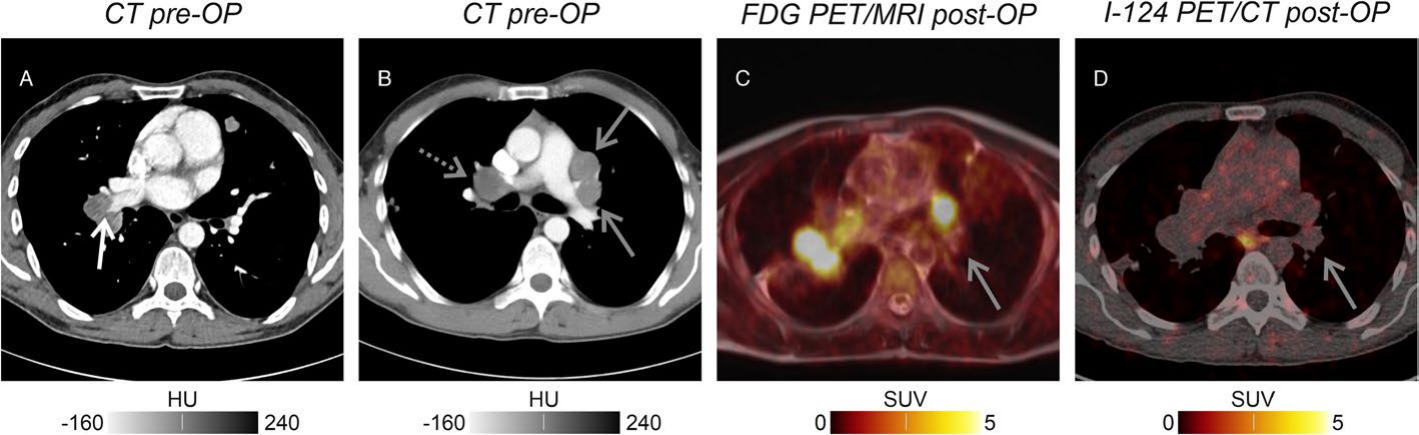
CT pulmonary angiogram (**A**, **B**) with exemplarily indicated filling defect of contrast media (white arrow) and bihilar metastases (solid and dashed grey arrows). ^18^F-FDG-PET/MRI (**C**) and ^124^I-PET/CT (**D**) after incomplete left hilar metastasectomy showing a partly resected radioiodine-negative left hilar metastasis (solid grey arrows)

A CT pulmonary angiogram directly after the initial V/P SPECT but preceding both metastasectomies revealed bilateral pulmonary artery compression due to bihilar metastases (Fig. 2B); however, also contrast media filling defects in segmental arteries were reported (Fig. 2A). In a follow-up ^18^F-FDG PET/MRI 2 months later, right hilar metastases were still visualised with intense glucose uptake, whereas left hilar metastases were partly resected (Fig. 2C). As these metastases did not show radioiodine uptake in ^124^I PET/CT (Fig. 2D), systemic tyrosine kinase inhibitor treatment was started. Pulmonary perfusion defects due to pulmonary artery compression by hilar metastases were finally diagnosed. Pulmonary embolism was deemed unlikely due to the left-sided post-operative normalisation, persistence of right-sided V/P mismatches and the lack of clinical symptoms.

## Conclusions

Pulmonary artery compression may mimic pulmonary artery embolism in lung ventilation/perfusion scintigraphy and should be considered in tumour patients with hilar metastases. Ventilation/perfusion mismatches are reported for bronchopulmonary cancer; these are rare pitfalls in lung scintigraphy but commonly unilaterally affect a complete lobe or lung. Following the presented case, also bilateral lobar, segmental and subsegmental mismatches in patients with hilar metastases from non-bronchopulmonary cancer entities should be carefully evaluated.

## Data Availability

The data sets generated and/or analysed during the current study are not publicly available due to privacy legislation but are available from the corresponding author on reasonable request.

## Abbreviations

MAA: ^99M^Tc-macro-aggregated albumin
PE: Pulmonary embolism
V/P: Ventilation/perfusion
VTE: Venous thromboembolism
